# Assessing hemorrhagic risks in combination therapy: implications of angiogenesis inhibitors and immune checkpoint inhibitors

**DOI:** 10.3389/fimmu.2025.1527570

**Published:** 2025-02-10

**Authors:** Yuhui Yang, Pingping Long, Ying Tuo, Xiaoxiao Wang

**Affiliations:** ^1^ Department of Pharmacy, Peking Union Medical College Hospital, Peking Union Medical College, Chinese Academy of Medical Sciences, Beijing, China; ^2^ Department of Pharmacy, Chongqing University Cancer Hospital, Chongqing, China; ^3^ School of Pharmacy and Bioengineering, Chongqing University of Technology, Chongqing, China

**Keywords:** immune checkpoint inhibitors, angiogenesis inhibitors, FDA adverse event reporting system (FAERS), hemorrhagic risks, cancer therapy, immunotherapy

## Abstract

**Objective:**

This study aims to evaluate the hemorrhage risk in solid tumor patients receiving angiogenesis inhibitors (AGIs), immune checkpoint inhibitors (ICIs), and their combination using the FDA Adverse Event Reporting System (FAERS) database.

**Methods:**

Data from Q1 2011 to Q4 2023 were extracted from the FAERS database for solid tumor patients treated with AGIs, ICIs, or their combination. A disproportionality analysis was conducted by calculating the reporting odds ratio (ROR) and corresponding 95% confidence interval (CI), as well as the Proportional Reporting Ratio (PRR), to identify potential safety signals. To assess whether the hemorrhage risk is higher with combination therapy compared to monotherapy, additive and multiplicative models were employed to evaluate the interactions between combination and single-agent treatments.

**Results:**

The combination of AGIs and ICIs significantly increased the risk of hemorrhagic adverse events, particularly tumor and pulmonary hemorrhage. Hemorrhagic events were common in females (50.97%) and older patients (aged 64+), frequently occurring within the first 30 days of treatment (38.11%). Gingival hemorrhage (ROR 3, PRR 418.9) and tumor hemorrhage (ROR 9.65, PRR 1893.36) were most common in the AGI group, while tumor hemorrhage (ROR 9.49, PRR 1350.78) and pulmonary hemorrhage (ROR 2.6, PRR 98.97) were prominent in the ICI group. In the combination group, esophageal variceal hemorrhage (ROR 40.72, PRR 2344.72) and tumor hemorrhage (ROR 19.31, PRR 1056.63) exhibited significantly increased risks Additive and multiplicative models indicated that the excess risk (RD_AB_ = 0.01025, P<0.001) and relative risk (RR_AB_ = 1.99277, P<0.001) of combination therapy were significantly higher than those of monotherapy, suggesting a positive interaction between the drugs that further increases the risk of hemorrhage.

**Conclusion:**

Our study demonstrates that the combination of AGIs and ICIs significantly raises the risk of hemorrhage, underscoring the urgent need for enhanced monitoring protocols in clinical practice to improve treatment efficacy and safety.

## 1 Introduction

In recent years, advancements in medical technology have significantly increased the survival time of cancer patients, with 53% surviving more than 10 years (1). The rise in cancer survival rates is primarily due to continuous advancements in anti-tumor therapies.

The development of drugs such as immune checkpoint inhibitors (ICIs) and angiogenesis inhibitors (AGIs) has enabled us to treat advanced cancers that are unresponsive to conventional chemotherapy, thereby extending survival. AGIs and ICIs have become key treatment options for various malignancies; however, their effectiveness is limited when used alone due to resistance development, lack of responsiveness, and frequent severe adverse reactions (2–6). The combination of ICIs and AGIs provides a multidimensional strategy for cancer treatment, offering a novel approach to overcoming tumor immune tolerance, enhancing immune efficacy, and improving the tumor microenvironment, showing great potential in enhancing cancer treatment outcomes (7, 8).

Currently, more than 80 combinations of anti-angiogenic and immunotherapy drugs are under evaluation (9). Although these agents have a low incidence of severe toxicity, they can cause drug-related adverse events that may lead to treatment interruptions, discontinuation, and negatively impact patient quality of life (10). Cancer patients inherently face a high risk of both thrombosis and hemorrhage (11), making hemorrhage prevention particularly challenging (12). The widespread use of these treatments has introduced additional challenges, notably an increased risk of hemorrhage (13), which is strongly associated with poor prognosis and high mortality (14, 15).

Both therapies may increase the risk of hemorrhage. AGIs exhibit strong anti-VEGF activity, and all AGIs carry a risk of hemorrhage. This may be due to vascular instability and loss of vessel integrity caused by reduced matrix deposition, leading to vessel rupture and thrombocytopenia (16). The most common hemorrhage manifestation is mild epistaxis (17). Additionally, direct antitumor activity may lead to cavitation in tumor regions containing dysplastic neovessels lacking robust and well-formed muscular structures, a condition believed to contribute to pulmonary hemorrhage, particularly in lung squamous cell carcinoma (18). Lastly, concurrent thrombocytopenia may exacerbate hemorrhage. In clinical trials of ICIs, most hemorrhagic events associated with these therapies are classified as serious adverse events. Hemorrhage can range from low-grade oozing to major spontaneous events, and even catastrophic hemorrhage. As hemorrhage can progress rapidly, inadequate management may be life-threatening (19).

Previous studies on the hemorrhage risk of AGIs and ICIs have mostly focused on single drug class risks, relying on clinical trial reports and case analyses, with limited real-world data. A systematic, large-scale evaluation of the hemorrhage risk associated with the combined use of AGIs and ICIs is currently lacking. Based on over 17 million real-world data entries from the FAERS, this study is the first to systematically evaluate the impact of AGIs, ICIs, and their combination on hemorrhage risks in solid tumor patients. The study offers an in-depth analysis of the risks associated with various types of hemorrhagic events, examining their temporal distribution and demographic factors. Additionally, using additive and multiplicative models, this study reveals for the first time that the combined use of AGIs and ICIs significantly increases hemorrhage risk, suggesting a synergistic effect in their drug interaction. These innovative findings fill the gap in the existing literature on the assessment of hemorrhage risk in combination therapy, providing important scientific evidence for clinical decision-making and risk management.

## 2 Methods

### 2.1 Data sources and preprocessing

**Figure 1:**
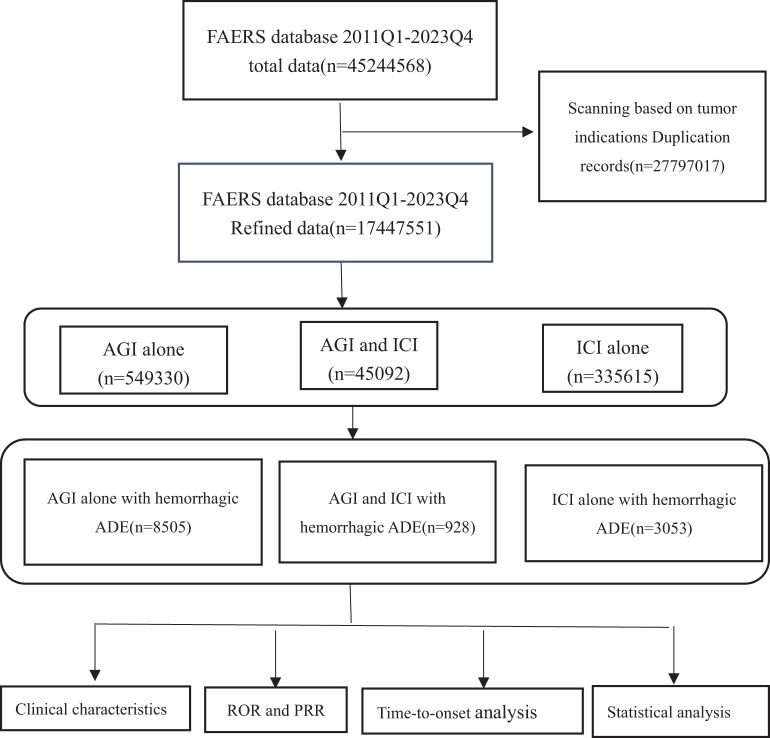
Flow chart showing the analysis process of the study. AGI, angiogenesis inhibitor; ICI, immune checkpoint inhibitor.

This study utilized FAERS data to analyze adverse reactions in patients receiving AGIs and ICIs the specific process is illustrated in **Figure 1**. The data were obtained from the FAERS database and specifically extracted from the FDA website (https://open.fda.gov/data/faers/). The FAERS database compiles spontaneous adverse event (AE) reports from healthcare professionals, manufacturers, and consumers globally. FAERS data files comprise seven datasets: patient demographics and administrative information (DEMO), drug/biologic information (DRUG), adverse events (REAC), patient outcomes (OUTC), report sources (RPSR), drug therapy start and end dates (THER), and indications (INDI). In accordance with FDA recommendations, duplicate records were removed before statistical analysis. If the CASEID was identical, the latest EVENT_DT was selected; if both CASEID and EVENT_DT were identical, the higher PRIMARYID was chosen.

The database spans from the first quarter of 2011 to the fourth quarter of 2023.

### 2.2 Inclusion criteria for drug data

The inclusion criteria for drug data are as follows: 1. For the AGIs group: the inclusion criteria involved suspected drugs containing AGIs (primary suspect, PS), with no ICIs included in the drug combination (secondary suspect, SS; concomitant drugs, C; interacting drugs, I). 2. For the ICIs group: the inclusion criteria involved suspected drugs containing ICIs (PS), with no AGIs included in the drug combination (SS, C, I). 3. For the combined angiogenesis inhibitor and immune checkpoint inhibitor group: the inclusion criteria involved suspected drugs containing AGIs (PS) + ICIs (SS, C, I) or ICIs (PS) + AGIs (SS, C, I). 4. Cases were excluded if the indication for drug use was not a solid tumor. 5. Cases were excluded if the time from drug initiation to symptom onset exceeded two years.

### 2.3 Target event and target drug classification

The target event was identified by the preferred term “hemorrhage” (Medical Dictionary for Regulatory Activities [MedDRA 26.1], MedDRA code 10055798). All hemorrhage events related to AGIs, ICIs, and their combination in the FAERS database were summarized in supplementary tables and categorized according to the MedDRA 26.1 System Organ Classifications (SOCs). The study drugs include two categories: the first category is AGIs, including bevacizumab, ramucirumab, aflibercept, sorafenib, regorafenib, cabozantinib, sunitinib, axitinib, nintedanib, lenvatinib, pazopanib, vandetanib, fruquintinib, and erdafitinib. The second category is ICIs, including atezolizumab, avelumab, cemiplimab, durvalumab, ipilimumab, nivolumab, pembrolizumab, dostarlimab, relatlimab, tremelimumab, retifanlimab, and toripalimab. Mapping was performed using multiple database fields, including generic names, brand names, and active ingredients.

### 2.4 Statistical analysis

Currently, disproportionality analysis (also known as case-noncase analysis) is widely used in pharmacovigilance studies for signal detection (20, 21). The Reporting Odds Ratio (ROR) and Proportional Reporting Ratio (PRR) are two commonly used disproportionality analysis methods in pharmacovigilance for identifying potential associations between drugs and adverse reactions. To obtain robust results, statistical shrinkage transformations were applied, and the corresponding formulas are as follows:


$$\begin{array}{l} \text{ROR}=\text{ad}/\text{bc}\\ 95\%\text{CI}={\text{e}}^{\text{ln}(\text{ROR})\pm 1.96(1/\text{a}+1/\text{b}+1/\text{c}+1/\text{d})\hat{\;}.5} \end{array}$$

The criteria for evaluation are lower limit of 95% CI>1, N≥3.


$$\begin{array}{c} \text{PRR}=\text{a}(\text{c}+\text{d})/\text{c}/(\text{a}+\text{b})\\ {\text{χ}}^{2}=[(\text{ad}-\text{bc})\hat{\;}2](\text{a}+\text{b}+\text{c}+\text{d})/[(\text{a}+\text{b})(\text{c}+\text{d})\\ (\text{a}+\text{c})(\text{b}+\text{d})]. \end{array}$$

The criteria for evaluation are PRR≥2, ${\text{χ}}^{2}$ ≥4, N≥3.

Equation: a, number of reports containing both the target drug and target adverse drug reaction; b, number of reports containing other adverse drug reaction of the target drug; c, number of reports containing the target adverse drug reaction of other drugs; d, number of reports containing other drugs and other adverse drug reactions. 95%CI, 95% confidence interval; N, the number of reports; χ^2^, chi-squared.

To determine whether the risk of hemorrhage is increased when AGIs and ICIs are used in combination compared to their use alone, additive and multiplicative models were used to assess the presence of interaction between combination therapy and monotherapy. They capture drug interactions in terms of absolute and relative risks, respectively, providing a more comprehensive risk assessment. Corresponding statistical tests were conducted to clarify the presence of drug interactions (22). The corresponding formulas are as follows:

#### 2.4.1 Additive model

Under the additive assumption, there is no interaction if the excess risk of A without B is the same as the excess risk of A with B:


$$\begin{array}{c} \text{risk}(\text{A},\neg \text{B})-\text{risk}(\neg \text{A},\neg \text{B})=\text{risk}(\text{A},\text{B})-\text{risk}(\neg \text{A},\text{B}),\;i.e.,\;RD_{\text{AB}}\\ =RD_{A}+RD_{B}. \end{array}$$

Under the additive assumption, if there is no interaction, the combined excess risk equals the sum of the excess risks associated with each drug used alone. When RD_AB_ > RD_A_ + RD_B_ (i.e., RD_AB_ - RD_A_ - RD_B_ > 0), there is a potential interaction between the combination therapy and an increased risk compared to the expected risk based on the individual drugs.

For a specific drug combination, the proportion of adverse events follows an approximately binomial distribution. The SAS program “proc genmod” was used to implement the additive model with an identity (identity-link) function and the multiplicative model with a log-linear (log-link) function. Suspicious drug-drug interactions were analyzed separately (23).


$$\begin{array}{c} \text{Event risk}=\alpha +\text{β}(\text{drug A})+\text{γ}(\text{drug B})+\text{δ}(\text{drug A and B})\\ +\text{other covariates}. \end{array}$$

The interaction measure is given by the coefficient δ, which measures the extent to which the combined use of A and B exceeds the predicted total risk of using A and B separately. Of particular interest is the statistical deviation of δ from 0, especially when δ > 0, indicating a positive interaction.

#### 2.4.2 Multiplicative model

When there is no interaction on a multiplicative scale, the relative risk associated with drug A is the same whether or not drug B is present. Formally,


$$\begin{array}{c} \frac{risk\left(A,\neg B\right)}{\text{risk}\left(\neg \text{A},\neg \text{B}\right)}=\frac{\text{risk}\left(\text{A},\text{B}\right)}{\text{risk}\left(\neg \text{A},\text{B}\right)}\underset{\;}{\Rightarrow }\frac{\text{risk}\left(\text{A},\text{B}\right)}{\text{risk}\left(\neg \text{A},\text{B}\right)}\\ =\frac{risk\left(A,\neg B\right)}{\text{risk}\left(\neg \text{A},\neg \text{B}\right)}\times \frac{risk\left(\neg A,B\right)}{\text{risk}\left(\neg \text{A},\neg \text{B}\right)} \end{array}$$

That is, RRAB = RRA × RRB. Under the assumption of no interaction, the relative risk associated with the drug combination is equal to the product of the relative risks of each drug used alone without the presence of the other drug. Therefore, if RRAB/(RRA × RRB) is statistically different from 1, there is evidence of interaction. Particularly, when this ratio is greater than 1, it indicates a positive interaction from a safety perspective. In this case, the relative risk associated with the combined use of the two drugs exceeds the product of the relative risks of each drug used alone.

In the framework of log-linear regression (e.g. logistic regression or Poisson regression), the formal statistical test for the interaction term can be implemented:


$$\begin{array}{c} \log(\text{event risk})=\text{α}+\text{β}(\text{drug A})+\text{γ}(\text{drug B})+\text{δ}(\text{drug A and B})\\ +\text{other covariates}. \end{array}$$

Whenever the coefficient δ is statistically significantly different from zero, there is evidence of interaction. When δ is greater than zero, it indicates a positive interaction, meaning that the combined event risk is greater than the predicted risk product of using each drug alone. When δ is less than zero, it indicates that the relative risk associated with the combined use of the two drugs is less than the product of the relative risks associated with each drug used alone. The exponent of δ, exp(δ), quantifies how much the relative risk of using A and B together exceeds the predicted relative risk of using A and B separately.

Data extraction and statistical analyses were performed using SAS (version 9.4), R (version 4.3.3), Excel 2020, and Origin 8.0 software.

## 3 Results

### 3.1 Descriptive analysis

From the first quarter of 2011 to the fourth quarter of 2023, a total of 45,244,568 reports were retrieved from the FAERS database. After removing duplicates and reports unrelated to solid tumor patients, 17,447,551 reports were included in the analysis. Detailed information is presented in **Table 1**. This dataset included 549,330 reports of angiogenesis inhibitor use alone, with 8,505 reports of hemorrhagic adverse events; 335,615 reports of ICIs used alone, with 3,053 reports of hemorrhagic adverse events; and 45,092 reports of combined AGIs and ICIs use, with 928 reports of hemorrhagic adverse events. Among hemorrhage-related adverse events, 90.93% of reports involving AGIs were from females, while 60.10% of reports involving ICIs were from males, and 35.13% of combination therapy reports were from males. Age distribution indicated that patients over 64 years were predominant in all treatment groups: 38.17% in the angiogenesis inhibitor group, 43.63% in the immune checkpoint inhibitor group, and 40.84% in the combination therapy group. Most reports, especially in the combination therapy group, were submitted by physicians, with over 80% (85.78%) of adverse drug event (ADE) reports provided by healthcare professionals, greatly enhancing the credibility of our analysis. The primary source countries of the reports were the United States, Japan, and China, with the highest number of reports originating from the United States. From 2017 to 2023, the number of reports gradually increased, with a notable rise in combination therapy reports in 2023, which reached 44.40%. The most commonly reported outcomes of adverse events included hospitalization or prolonged hospital stays, other serious events, and death.

**Table 1:** Baseline characteristics of hemorrhagic reports associated with angiogenesis inhibitors, immune checkpoint inhibitors and combination therapy from 2011 to 2023.

Characteristics	Report number, N (%)		
	AGI	ICI	Combination therapy
Number of reports	8505	3053	928
Gender, n (%)			
Female	7734 (90.93)	1031 (33.77)	473 (50.97)
Male	636 (7.48)	1835 (60.10)	326 (35.13)
Unknown or missing	135 (1.59)	187 (6.13)	129 (13.90)
Age (years), n (%)			
<18	19 (0.22)	6 (0.20)	0
18≤and ≤ 64	2638 (31.02)	1018 (33.34)	309 (33.30)
>64	3246 (38.17)	1332 (43.63)	379 (40.84)
Unknown or missing	2602 (30.59)	697 (22.83)	240 (25.86)
Serious outcome, n (%)			
Death	1187 (13.96)	446 (14.61)	139 (14.98)
Life-threatening	239 (2.81)	98 (3.21)	28 (3.02)
Hospitalization	2740 (32.22)	666 (21.81)	433 (46.66)
Disability	113 (1.33)	26 (0.85)	5 (0.54)
Others	4226 (49.69)	1817 (59.52)	323 (34.81)
Reported Countries (Top five), n (%)			
United States	3629 (42.67)	788 (25.81)	204 (22.09)
Japan	1440 (16.93)	928 (30.40)	187 (20.15)
China	496 (5.83)	145 (4.70)	101 (10.88)
Canada	378 (4.44)		
France	275 (3.23)	189 (6.19)	65 (7.00)
Germany		179 (5.86)	
United Kingdom			60 (6.47)
Reported Person, n (%)			
Physician	3147 (37.00)	1402 (45.92)	665 (71.66)
Pharmacist	498 (5.86)	146 (4.78)	22 (2.37)
Other health-professional	569 (6.69)	332 (10.87)	109 (11.75)
Consumer	3261 (38.34)	719 (23.55)	116 (12.50)
Unknown	1030 (12.11)	454 (14.87)	16 (1.73)
Reporting year, n (%)			
2011	118 (1.39)	17 (0.56)	1 (0.11)
2012	278 (3.27)	27 (0.88)	2 (0.22)
2013	456 (5.36)	39 (1.28)	0
2014	553 (6.50)	44 (1.44)	0
2015	845 (9.94)	115 (3.77)	2 (0.22)
2016	700 (8.23)	182 (5.96)	0
2017	908 (10.68)	257 (8.42)	7 (0.75)
2018	890 (10.46)	388 (12.71)	15 (1.62)
2019	885 (10.41)	381 (12.48)	44 (4.74)
2020	790 (9.29)	413 (13.53)	95 (10.24)
2021	738 (8.68)	363 (11.89)	122 (13.15)
2022	639 (7.51)	391 (12.81)	228 (24.57)
2023	705 (8.29)	436 (14.28)	412 (44.40)

### 3.2 A statistical analysis of hemorrhagic adverse events across different treatment groups

**Table 2** ranks the PT signals of AGIs, ICIs, and their combined use (AGIs + ICIs) based on occurrence frequency. Among AGIs, the most common adverse events were gingival bleeding (345 cases, ROR 3, PRR 418.9) and tumor hemorrhage (321 cases, ROR 9.65, PRR 1893.36). For ICIs, tumor hemorrhage (211 cases, ROR 9.49, PRR 1350.78) and pulmonary hemorrhage (106 cases, ROR 2.6, PRR 98.97) were the most frequent. When AGIs and ICIs were combined, esophageal variceal hemorrhage (67 cases, ROR 40.72, PRR 2344.72) and tumor hemorrhage (64 cases, ROR 19.31, PRR 1056.63) were the most prominent. Additionally, gingival bleeding, pulmonary hemorrhage, and oral hemorrhage were common across all three treatment scenarios, particularly with increased risks in combination therapy. Tumor hemorrhage represented a significant risk across all treatment groups, with 321 cases in the angiogenesis inhibitor group, 211 cases in the ICIs group, and 64 cases in the combination group. Gingival bleeding was common in both the angiogenesis inhibitor and combination groups; pulmonary hemorrhage occurred in all three groups; gastric hemorrhage was more frequent in the angiogenesis inhibitor and combination groups; and oral hemorrhage frequently appeared in both the angiogenesis inhibitor and combination groups. Notably, the risk of esophageal variceal hemorrhage was greatest in combination therapy, with 67 cases reported. These data indicate that the combined use of AGIs and ICIs significantly increases the risk of various types of hemorrhagic adverse reactions, particularly esophageal variceal hemorrhage and tumor hemorrhage.

**Table 2:** Top 10 hemorrhagic signals for each treatment group, sorted by frequency.

PT	SOC	Freq	ROR (95%)	PRR (X^2^)
AGI (Sorted by frequency)				
Gingival bleeding	Gastrointestinal disorders	345	3 (2.69-3.35)	3 (418.9)
Tumour hemorrhage	Neoplasms benign, malignant and unspecified (incl cysts and polyps)	321	9.65 (8.51-10.94)	9.64 (1893.36)
Eye hemorrhage	Eye disorders	318	3.05 (2.72-3.43)	3.05 (399.01)
Gastric hemorrhage	Gastrointestinal disorders	189	2.47 (2.13-2.86)	2.47 (152.9)
Mouth hemorrhage	Gastrointestinal disorders	187	2.94 (2.53-3.42)	2.94 (218.44)
Retinal hemorrhage	Eye disorders	172	4.18 (3.56-4.9)	4.18 (365.83)
Pulmonary hemorrhage	Respiratory, thoracic and mediastinal disorders	162	2.46 (2.1-2.89)	2.46 (129.96)
Oesophageal varices hemorrhage	Gastrointestinal disorders	156	8.78 (7.34-10.48)	8.77 (835.99)
Haemorrhoidal hemorrhage	Gastrointestinal disorders	145	2.97 (2.5-3.52)	2.97 (172.32)
Vitreous hemorrhage	Eye disorders	102	6.31 (5.1-7.81)	6.31 (378.45)
ICIs (Sorted by frequency)				
Tumour hemorrhage	Neoplasms benign, malignant and unspecified (incl cysts and polyps)	211	9.49 (8.19-11)	9.49 (1350.78)
Pulmonary hemorrhage	Respiratory, thoracic and mediastinal disorders	106	2.6 (2.14-3.16)	2.6 (98.97)
Enterocolitis haemorrhagic	Gastrointestinal disorders	70	11.02 (8.51-14.27)	11.02 (524.21)
Small intestinal hemorrhage	Gastrointestinal disorders	47	3.9 (2.9-5.24)	3.9 (94.02)
Intracranial tumour haemorrhage	Neoplasms benign, malignant and unspecified (incl cysts and polyps)	38	13.27 (9.29-18.97)	13.27 (342.11)
Gastritis haemorrhagic	Gastrointestinal disorders	32	3.29 (2.3-4.7)	3.29 (47.9)
Cerebellar haemorrhage	Nervous system disorders	19	2.47 (1.56-3.92)	2.47 (15.87)
Hepatic haemorrhage	Hepatobiliary disorders	17	3.9 (2.38-6.39)	3.9 (34.12)
Adrenal haemorrhage	Endocrine disorders	10	5.26 (2.74-10.08)	5.26 (31.25)
Laryngeal haemorrhage	Respiratory, thoracic and mediastinal disorders	8	12.36 (5.71-26.76)	12.36 (67.23)
AGI+ICIs (Sorted by frequency)				
Oesophageal varices haemorrhage	Gastrointestinal disorders	67	40.72 (31.65-52.38)	40.66 (2344.72)
Cerebral haemorrhage	Nervous system disorders	65	2.51 (1.96-3.2)	2.5 (58.39)
Tumour haemorrhage	Neoplasms benign, malignant and unspecified (incl cysts and polyps)	64	19.31 (15.02-24.82)	19.28 (1056.63)
Upper gastrointestinal haemorrhage	Gastrointestinal disorders	64	3.91 (3.06-5.01)	3.91 (137.23)
Gingival bleeding	Gastrointestinal disorders	26	2.6 (1.77-3.83)	2.6 (25.45)
Pulmonary hemorrhage	Respiratory, thoracic and mediastinal disorders	22	3.92 (2.58-5.97)	3.92 (47.38)
Haemorrhagic stroke	Nervous system disorders	18	3.02 (1.9-4.81)	3.02 (24.16)
Gastric hemorrhage	Gastrointestinal disorders	17	2.6 (1.61-4.18)	2.6 (16.58)
Stoma site hemorrhage	Injury, poisoning and procedural complications	16	9.46 (5.76-15.53)	9.46 (118.1)
Mouth hemorrhage	Gastrointestinal disorders	15	2.72 (1.64-4.52)	2.72 (16.19)

**Table 3** ranks the PT signals of AGIs, ICIs, and their combination (AGIs + ICIs) by ROR. In the AGIs group, lymph node hemorrhage (ROR 17.09) and fragmented hemorrhage (ROR 14.77) were the most significant, followed by high ROR events such as intestinal variceal hemorrhage (ROR 13.67), hemorrhagic tumor necrosis (ROR 10.25), and tumor hemorrhage (ROR 9.65). In the ICIs group, intracranial tumor hemorrhage (ROR 13.27) and laryngeal hemorrhage (ROR 12.36) were the most notable, along with hemorrhagic colitis (ROR 11.02) and tumor hemorrhage (ROR 9.49). In the combination of AGIs and ICIs, injection site hemorrhage (ROR 53.24) and esophageal variceal hemorrhage (ROR 40.72) were particularly prominent, with other high ROR events including tumor hemorrhage (ROR 19.31) and gastric variceal hemorrhage (ROR 19.3). Tumor hemorrhage showed high ROR values across all three drug groups: AGIs (ROR 9.65), ICIs (ROR 9.49), and combination therapy (ROR 19.31), indicating that tumor hemorrhage is a significant risk in all treatment scenarios.

### 3.3 Time to onset of hemorrhage

**Figure 2:**
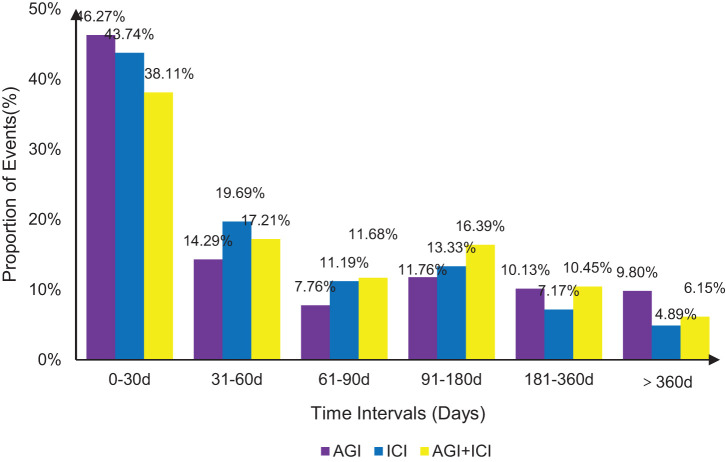
Time to onset of hemorrhagic events by treatment group.

**Figure 2** illustrates the time distribution of hemorrhagic adverse events induced by AGIs, ICIs, and their combination (AGIs + ICIs), with 2,695, 1,493, and 488 reports included, respectively. Within the first 30 days, the proportion of hemorrhage events was highest, at 46.27% for AGIs, 43.74% for ICIs, and 38.11% for the combination group. Over time, the proportion of hemorrhage events gradually declined in all groups. Between 31 and 60 days, the ICI group exhibited the highest proportion of hemorrhage events at 19.69%, while the AGIs group and the combination group had 14.29% and 17.21%, respectively. Between 91 and 180 days, the combination group demonstrated a significantly higher proportion of hemorrhage events at 16.39% compared to either the AGIs or ICIs groups alone. In the periods of 181-360 days and beyond 360 days, the proportion of hemorrhage events in all groups remained below 10%. Overall, the combination of AGIs and ICIs was associated with an increased risk of hemorrhage across multiple time intervals.

### 3.4 Use additive and multiplicative models to assess drug interactions

**Table 2** indicates that tumor hemorrhage poses a significant risk across all treatment scenarios, while pulmonary hemorrhage is prevalent in all three treatment types. **Table 3** demonstrates that tumor hemorrhage consistently displays high Reporting Odds Ratios (ROR) across the three drug categories. Consequently, the raw data for all hemorrhagic events, including tumor hemorrhage and pulmonary hemorrhage, were compiled. From 2011 to 2023, the FAERS database recorded 17,447,551 adverse event reports. Of these, 549,330 reports pertained to anti-angiogenic inhibitors (AGIs), 335,615 reports pertained to immune checkpoint inhibitors (ICIs), and 45,092 reports involved the combination of AGIs and ICIs. The detailed results are presented in **Table 4**.

**Table 3:** Top 10 hemorrhagic signals for each treatment group, Sorted by ROR.

PT	SOC	Freq	ROR (95%)	PRR (X^2^)
AGI (Sorted by ROR)				
Lymph node hemorrhage	Blood and lymphatic system disorders	5	17.09 (5.73-50.99)	17.09 (48.69)
Splinter hemorrhages	Skin and subcutaneous tissue disorders	12	14.77 (7.42-29.39)	14.77 (104.05)
Intestinal varices hemorrhage	Gastrointestinal disorders	4	13.67 (4.21-44.4)	13.67 (32.52)
Haemorrhagic tumour necrosis	Neoplasms benign, malignant and unspecified (incl cysts and polyps)	5	10.25 (3.73-28.21)	10.25 (31.32)
Tumour hemorrhage	Neoplasms benign, malignant and unspecified (incl cysts and polyps)	321	9.65 (8.51-10.94)	9.64 (1893.36)
Tracheal hemorrhage	Injury, poisoning and procedural complications	24	8.9 (5.65-14.01)	8.89 (130.45)
Oesophageal varices hemorrhage	Gastrointestinal disorders	156	8.78 (7.34-10.48)	8.77 (835.99)
Scleral hemorrhage	Eye disorders	11	8.68 (4.44-16.94)	8.68 (58.27)
Hemorrhage coronary artery	Cardiac disorders	3	8.39 (2.34-30.07)	8.39 (15.34)
Haemobilia	Hepatobiliary disorders	28	7.9 (5.22-11.97)	7.9 (134.3)
ICIs (Sorted by ROR)				
Intracranial tumour hemorrhage	Neoplasms benign, malignant and unspecified (incl cysts and polyps)	38	13.27 (9.29-18.97)	13.27 (342.11)
Laryngeal hemorrhage	Respiratory, thoracic and mediastinal disorders	8	12.36 (5.71-26.76)	12.36 (67.23)
Enterocolitis haemorrhagic	Gastrointestinal disorders	70	11.02 (8.51-14.27)	11.02 (524.21)
Tumour hemorrhage	Neoplasms benign, malignant and unspecified (incl cysts and polyps)	211	9.49 (8.19-11)	9.49 (1350.78)
Acute haemorrhagic ulcerative colitis	Gastrointestinal disorders	3	7.65 (2.27-25.74)	7.65 (15.08)
Stomatitis haemorrhagic	Gastrointestinal disorders	5	6.71 (2.64-17.05)	6.71 (21.46)
Pituitary hemorrhage	Endocrine disorders	7	6.15 (2.81-13.48)	6.15 (26.96)
Adrenal hemorrhage	Endocrine disorders	10	5.26 (2.74-10.08)	5.26 (31.25)
Small intestinal hemorrhage	Gastrointestinal disorders	47	3.9 (2.9-5.24)	3.9 (94.02)
Hepatic hemorrhage	Hepatobiliary disorders	17	3.9 (2.38-6.39)	3.9 (34.12)
AGI+ICIs (Sorted by ROR)				
Administration site hemorrhage	General disorders and administration site conditions	4	53.24 (18.71-151.44)	53.23 (180.15)
Oesophageal varices hemorrhage	Gastrointestinal disorders	67	40.72 (31.65-52.38)	40.66 (2344.72)
Tumour hemorrhage	Neoplasms benign, malignant and unspecified (incl cysts and polyps)	64	19.31 (15.02-24.82)	19.28 (1056.63)
Gastric varices hemorrhage	Gastrointestinal disorders	5	19.3 (7.86-47.38)	19.3 (82.61)
Haemobilia	Hepatobiliary disorders	6	17.68 (7.8-40.07)	17.68 (90.26)
Hepatic hemorrhage	Hepatobiliary disorders	7	11.65 (5.49-24.7)	11.64 (66.12)
Spinal cord hemorrhage	Nervous system disorders	3	10.43 (3.31-32.84)	10.43 (24.91)
Stoma site hemorrhage	Injury, poisoning and procedural complications	16	9.46 (5.76-15.53)	9.46 (118.1)
Skin ulcer hemorrhage	Skin and subcutaneous tissue disorders	3	9.41 (2.99-29.59)	9.41 (22.02)
Oesophageal hemorrhage	Gastrointestinal disorders	9	8.98 (4.64-17.38)	8.98 (62.33)

**Table 4:** Risk proportions of specific adverse events following the use of AGIs, ICIs, and their combination (AGIs + ICIs).

ADE	Combination (AGIs and ICIs)	AGIs Only	ICIs Only	Neither AGIs nor ICIs
All hemorrhage ADE	928/45092	8505/549330	3053/335615	171049/16562606
Tumour hemorrhage	64/45092	321/549330	211/335615	1281/16562606
Pulmonary hemorrhage	22/45092	162/549330	106/335615	2166/16562606

All hemorrhage, tumor hemorrhage, and pulmonary hemorrhage were preliminarily screened as potential signals, and the results are shown in **Table 5**. The signal testing results are shown in **Table 6**.

**Table 5:** Signal detection results of additive and multiplicative models.

ADE	Additive model			Multiplicative model		
	RD_AB_	RD_A_+RD_B_	Difference	RR_AB_	RR_A_×RR_B_	Ratio
All hemorrhage ADE	0.01025	0.00392	0.00633	1.99277	1.32051	1.50909
Tumour hemorrhage	0.00134	0.00106	0.00028	18.35101	61.41464	0.29881
Pulmonary hemorrhage	0.00036	0.00035	7.93E-06	3.73073	5.44611	0.68503

RD_AB_, Excess Risk of Combined Use of AGIs and ICIs; RD_A_, Excess Risk of AGIs Alone; RD_B_, Excess Risk of ICIs Alone.

**Table 6:** Statistical results of additive and multiplicative models for hemorrhagic events.

ADE	Additive model (α=0.05)		Multiplicative model (α=0.05)	
	δ	P value	exp(δ)	P value
All hemorrhage ADE	0.0103	<0.001	1.9927	<0.001
Tumour hemorrhage	0.0013	<.0001	18.3513	<.0001
Pulmonary hemorrhage	0.0004	0.0006	3.7307	<.0001

**Tables 5** and **6** indicate that the combination of AGIs and ICIs significantly elevates the risk of hemorrhagic adverse events. Both additive and multiplicative models for hemorrhagic events demonstrated significant interactions, with an excess risk (RD_AB_) of 0.01025 (P < 0.001) and a relative risk (RR_AB_) of 1.99277 (P < 0.001), both exceeding the combined risks from monotherapy (RD_A_ + RD_B_ and RR_A_ + RR_B_). Tumor hemorrhage and pulmonary hemorrhage exhibited similar trends, particularly tumor hemorrhage, which had an excess risk (RD_AB_) of 0.00134 (P < 0.0001) and a relative risk (RR_AB_) of 18.35101 (P < 0.0001), significantly surpassing the risks from monotherapy (RR_A_ + RR_B_). For pulmonary hemorrhage, the excess risk (RD_AB_) was 0.00036 (P = 0.0006), and the relative risk (RR_AB_) was 3.73073 (P < 0.0001). These results confirm that the combination of AGIs and ICIs significantly raises the risk of specific hemorrhagic adverse events, highlighting a notable drug interaction.

## 4 Discussion

Anti-angiogenic drugs are one of the few combination partners that have been clinically proven to significantly enhance the efficacy of immune checkpoint inhibitors. This benefit has been validated in pivotal phase III trials across various cancer types, with some yielding practice-changing results (24). Since 2018, when Choueiri et al. first reported the efficacy of avelumab combined with axitinib for previously untreated advanced renal cell carcinoma (25), more solid tumor patients have begun using the treatment strategy that combines angiogenesis inhibitors with immune checkpoint inhibitors (26, 27).

The combination of angiogenesis inhibitors and immune checkpoint inhibitors not only results in additive efficacy but also generates synergistic effects. Anti-angiogenic drugs can block multiple immunosuppressive effects of VEGF and induce various vascular-regulating effects, such as vascular normalization leading to increased blood flow and perfusion within the tumor and inhibition of endothelial cell apoptosis effects on T cells. AGIs can shift the tumor microenvironment from immune suppression to immune activation, thereby enhancing anti-tumor immunity (28, 29). Inhibiting the VEGF-VEGFR axis has several beneficial effects that can enhance the efficacy of ICIs in principle (30–32). VEGF can also directly affect immune cell function and impair optimal anti-tumor immunity (33). The presence of synergistic effects further enhances therapeutic outcomes.

However, everything has two sides; while synergistic effects are produced, the likelihood of adverse reactions may also increase. It has been demonstrated that combination therapies lead to higher rates of irAEs (34). Previous studies have found that anti-angiogenic drugs, immune checkpoint inhibitors, and their combination increase the risk of hemorrhage. Hemorrhage can lead to severe consequences, warranting increased attention.

Anti-angiogenic drugs, when used alone, pose a significant risk of hemorrhage. All anti-angiogenic drugs carry a heightened risk of hemorrhage, which can even be fatal (17). In a phase II clinical trial of bevacizumab for non-small cell lung cancer, 6 patients (9%) experienced life-threatening pulmonary hemorrhage and/or hemoptysis, 4 of which were fatal (35). The high incidence of pulmonary hemorrhage in lung tumors may be related to bevacizumab’s efficacy in this environment, as many lung tumors exhibit necrosis or central cavitation, or are located near major blood vessels (36). Similarly, in a phase II trial of sunitinib for metastatic non-small cell lung cancer, two patients suffered fatal pulmonary hemorrhage (26). Vascular endothelial growth factor (VEGF) plays a crucial role in endothelial cell proliferation, survival, and maintaining vascular integrity (37). Inhibiting VEGF disrupts the regenerative capacity of endothelial cells, leading to vascular defects, which exposes the basement membrane and can result in thrombosis or hemorrhage (38).

Hemorrhage induced by immune checkpoint inhibitors can range from low-grade oozing to major spontaneous bleeding, or even catastrophic hemorrhage. Since hemorrhage can progress rapidly, improper management may be life-threatening (19). In a study of immune checkpoint inhibitors for advanced non-small cell lung cancer involving 83 patients, 6 cases (7.2%) of gastrointestinal hemorrhage, bronchial hemorrhage, and cerebral hemorrhage were reported (39). ICs can cause various immune-related adverse events, such as acquired hemophilia and acquired thrombotic thrombocytopenic purpura, potentially resulting in uncontrollable hemorrhage (40, 41). Mengting Chen et al. also found that immune checkpoint inhibitors increase the risk of hemorrhage (19).

Combination therapy warrants particular attention due to its hemorrhage risk. In 2022, Shijubou Naoki et al. reported a case of diffuse alveolar hemorrhage following combined use of atezolizumab and bevacizumab; in two clinical studies involving the combination, approximately 7% of participants experienced gastrointestinal hemorrhage (42). Tianqi Gu et al.’s research confirmed that combination therapy is an independent risk factor for hypertension and gastrointestinal hemorrhage (43).

To our knowledge, this is the first study to systematically assess the impact of angiogenesis inhibitors, immune checkpoint inhibitors, and their combination on the risk of hemorrhage in solid tumor patients using the FAERS database. The key findings are as follows:

Among hemorrhagic adverse events caused by anti-angiogenic drugs, females accounted for 90.93%, significantly higher than males. This suggests a potential gender difference in the use of anti-angiogenic drugs. However, there is currently a lack of gender-specific research on hemorrhage as an adverse event associated with anti-angiogenic drugs. In hemorrhagic adverse events caused by immune checkpoint inhibitors, females accounted for 33.77%. Our findings align with those of Mengting Chen et al., who reported that the frequency of hemorrhage reports is significantly lower in females compared to males (19). In combination therapy reports, females accounted for 50.97%, higher than males at 35.13%. This could be related to the higher likelihood of hemorrhage in females using anti-angiogenic drugs, or it could be associated with differences in the usage range of these drugs and gender differences in tumor incidence (44). Further research is needed to determine the impact of gender on hemorrhagic adverse events. In the age distribution results for anti-angiogenic drugs, immune checkpoint inhibitors, and their combination, the age group over 64 had the highest proportion, which may be related to the high incidence of most cancers occurring in individuals over 45 years old (45). Among hemorrhagic adverse events, the highest proportion occurred within 0-30 days of treatment, at 46.27%, 43.74%, and 38.11%, respectively. This indicates that clinical practice should closely monitor patients for hemorrhage within 30 days of treatment to avoid life-threatening outcomes. The excess risk and relative risk of combination therapy are significantly higher than those of monotherapy, suggesting a notable interaction between AGIs and ICs that increases the risk of hemorrhagic adverse events. Analysis of additive and multiplicative models indicates that the excess risk (RD_AB_) and relative risk (RR_AB_) of combination therapy are significantly higher than the combined risks of monotherapy, particularly for tumor and pulmonary hemorrhage. The hemorrhage risks in combination therapy are not merely additive toxicities but result from synergistic drug enhancement. Therefore, in clinical practice, patients undergoing combination therapy should be closely monitored, especially during early and mid-treatment phases, to ensure timely identification and effective management of potential hemorrhagic events.

However, this research has certain limitations. The data were sourced from the FAERS database, which has inherent limitations, including varying data quality and potential biases arising from differences among reporters. The reports lack detailed patient information, including drug dosage, treatment duration, pre-existing health conditions, and drug interactions, which limits the scope of further analysis. Additionally, the reporting frequency of adverse reactions varies among different drugs, necessitating more specific analyses to understand the hemorrhage risk of individual drugs. This study also lacks data on how different cancer types affect bleeding risk. Each AGI and ICI has distinct immunomodulatory effects, and the populations that benefit from these treatments may vary significantly (46). Future research will need well-designed clinical trials to determine optimal combination strategies for AGIs and ICIs, including appropriate dosages, dosing sequences, and therapeutic index optimization. Additionally, predictive biomarker studies will enable more effective assessment of adverse reaction risks, optimizing treatment strategies for patients at risk of bleeding or toxicity.

## 5 Conclusion

Although we minimized bias and confounding factors through rigorous data processing and statistical methods, including ROR, PRR, and additive and multiplicative interaction models, the spontaneous nature of the FAERS data and the lack of detailed clinical information mean that this study primarily suggests an association rather than establishing causality. Therefore, the results should be regarded as preliminary evidence that the combined use of AGIs and ICIs may increase hemorrhage risk. Our pharmacovigilance study indicates that the combined use of AGIs and ICIs significantly increases the risk of specific hemorrhagic adverse events, such as pulmonary hemorrhage and tumor bleeding, with a clear drug interaction. Future studies should further validate these findings through prospective cohort studies or randomized controlled trials to establish causality, and explore the appropriate dosage and timing of combination therapy to optimize the synergistic effect and minimize hemorrhagic toxicity. Additionally, there is an urgent need for real-world studies to validate these findings and assist healthcare providers in closely monitoring potential hemorrhage risks in the early stages of treatment.

## Data Availability

The original contributions presented in the study are included in the article/supplementary material. Further inquiries can be directed to the corresponding author.

## References

[1] Kimberly DM Leticia N Theresa D Angela BM K Robin Y Ahmedin J. Cancer treatment and survivorship statistics. CA Cancer J Clin. (2022) 72(5). doi: 10.3322/caac.21731

[2] Jun-Yan L Yu-Pei C Ying-Qin L Na L Jun M. Chemotherapeutic and targeted agents can modulate the tumor microenvironment and increase the efficacy of immune checkpoint blockades. (2021) 20(1). doi: 10.1186/s12943-021-01317-7 PMC7863268 33541368

[3] Upadhaya S Neftelino S Hodge J Oliva C Campbell J Yu J. Combinations take centre stage in PD1/PDL1 inhibitor clinical trials. Nat Rev Drug Discov. (2021) 20(3):168–9. doi: 10.1038/d41573-020-00204-y 33177720

[4] Syn N Teng M Mok T Soo R. *De-novo* and acquired resistance to immune checkpoint targeting. Lancet Oncol. (2017) 18(12):e731–e41. doi: 10.1016/S1470-2045(17)30607-1 29208439

[5] Huang Y Goel S Duda D Fukumura D Jain R. Vascular normalization as an emerging strategy to enhance cancer immunotherapy. Cancer Res. (2013) 73 2943–8. doi: 10.1158/0008-5472.CAN-12-4354 PMC3655127 23440426

[6] Bergers G Hanahan D. Modes of resistance to anti-angiogenic therapy. Nat Rev Cancer. (2008) 8(8):592–603. doi: 10.1038/nrc2442 18650835 PMC2874834

[7] Wang C Qiao W Jiang Y Zhu M Shao J Wang T. The landscape of immune checkpoint inhibitor plus chemotherapy versus immunotherapy for advanced non-small-cell lung cancer: A systematic review and meta-analysis. J Cell Physiol. (2020) 235(5):4913–27. doi: 10.1002/jcp.v235.5 PMC7028135 31693178

[8] Galluzzi L Humeau J Buqué A Zitvogel L Kroemer G. Immunostimulation with chemotherapy in the era of immune checkpoint inhibitors. Nat Rev Clin Oncol. (2020) 17(12):725–41. doi: 10.1038/s41571-020-0413-z 32760014

[9] Zowi RH Elisabeth JMH Judy R Patrycja N-S Arjan WG. Anti-angiogenic agents - overcoming tumour endothelial cell anergy and improving immunotherapy outcomes. Nat Rev Clin Oncol. (2021) 18(8). doi: 10.1038/s41571-021-00496-y 33833434

[10] Naidoo J Page D Li B Connell L Schindler K Lacouture M. Toxicities of the anti-PD-1 and anti-PD-L1 immune checkpoint antibodies. Ann Oncol. (2015) 26(12):2375–91. doi: 10.1093/annonc/mdv383 PMC6267867 26371282

[11] D JP. Hematologic abnormalities in patients with nonhematologic Malignancies. Hematology/oncology Clinics of North America. (1987) 1(2):281. doi: 10.1016/S0889-8588(18)30676-2 3308824

[12] Elice F Jacoub J Rickles F Falanga A Rodeghiero F. Hemostatic complications of angiogenesis inhibitors in cancer patients. Am J Hematol. (2008) 83(11):862–70. doi: 10.1002/ajh.v83:11 18819092

[13] Jose P Tien P. Management of bleeding in patients with advanced cancer. Oncologist. (2004) 9 (5). doi: 10.1634/theoncologist.9-5-561 15477642

[14] Dawn S Niamh L Mike M Jecko T. Management of bleeding and procedures in patients on antiplatelet therapy. Blood Rev. (2019) 39(5). doi: 10.1016/j.blre.2019.100619 31648803

[15] Sergio B Davide C Stefan J Dominick JA. Bleeding after antiplatelet therapy for the treatment of acute coronary syndromes: a review of the evidence and evolving paradigms. (2019) 18(2). doi: 10.1080/14740338.2019.1680637 31623473

[16] Inai T Mancuso M Hashizume H Baffert F Haskell A Baluk P. Inhibition of vascular endothelial growth factor (VEGF) signaling in cancer causes loss of endothelial fenestrations, regression of tumor vessels, and appearance of basement membrane ghosts. The American Journal of Pathology. (2004) 165(1):35–52. doi: 10.1016/S0002-9440(10)63273-7 15215160 PMC1618540

[17] Watson N Al-Samkari H. Thrombotic and bleeding risk of angiogenesis inhibitors in patients with and without Malignancy. J Thromb Haemost. (2021) 19(8):1852–63. doi: 10.1111/jth.15354 33928747

[18] Johnson D Fehrenbacher L Novotny W Herbst R Nemunaitis J Jablons D. Randomized phase II trial comparing bevacizumab plus carboplatin and paclitaxel with carboplatin and paclitaxel alone in previously untreated locally advanced or metastatic non-small-cell lung cancer. J Clin Oncol. (2004) 22(11):2184–91. doi: 10.1200/JCO.2004.11.022 15169807

[19] Mengting C Zhichao H Jianhong Z Shan Y Siyuan G Jie W. Hemorrhage profile associated with immune checkpoint inhibitors: a systematic review and a real-world study based on the FAERS database. (2024) 23(4). doi: 10.1080/14740338.2024.2327504 38556702

[20] Hou Y Ye X Wu G Cheng G Du X He J. A comparison of disproportionality analysis methods in national adverse drug reaction databases of China. Expert Opin Drug Saf. (2014) 13(7):853–7. doi: 10.1517/14740338.2014.915938 24918197

[21] Ang P Chen Z Chan C Tai B. Data mining spontaneous adverse drug event reports for safety signals in Singapore - a comparison of three different disproportionality measures. Expert Opin Drug Saf. (2016) 15(5):583–90. doi: 10.1517/14740338.2016.1167184 26996192

[22] Lina-Marcela D-G Boel B Helga W Rickard S Daniel R. Understanding interactions between risk factors, and assessing the utility of the additive and multiplicative models through simulations. PLoS One. (2021) 16(4). doi: 10.1371/journal.pone.0250282 PMC8075235 33901204

[23] Thakrar B Grundschober S Doessegger L. Detecting signals of drug-drug interactions in a spontaneous reports database. Br J Clin Pharmacol. (2007) 64(4):489–95. doi: 10.1111/j.1365-2125.2007.02900.x PMC2048563 17506784

[24] Kuo H Khan K Kerbel R. Antiangiogenic-immune-checkpoint inhibitor combinations: lessons from phase III clinical trials. Nat Rev Clin Oncol. (2024) 21(6):468–82. doi: 10.1038/s41571-024-00886-y 38600370

[25] Larkin J Oya M Martignoni M Thistlethwaite F Nathan P Ornstein M. Avelumab plus axitinib as first-line therapy for advanced renal cell carcinoma: long-term results from the JAVELIN renal 100 phase ib trial. Oncologist. (2023) 28(4):333–40. doi: 10.1093/oncolo/oyac243 PMC10078905 36576173

[26] Socinski M Novello S Brahmer J Rosell R Sanchez J Belani C. Multicenter, phase II trial of sunitinib in previously treated, advanced non-small-cell lung cancer. J Clin Oncol. (2008) 26(4):650–6. doi: 10.1200/JCO.2007.13.9303 PMC3559017 18235126

[27] Albiges L Powles T Staehler M Bensalah K Giles R Hora M. Updated european association of urology guidelines on renal cell carcinoma: immune checkpoint inhibition is the new backbone in first-line treatment of metastatic clear-cell renal cell carcinoma. Eur Urol. (2019) 76(2):151–6. doi: 10.1016/j.eururo.2019.05.022 31151678

[28] Bo Z Baorui T Yitong L Chenhe Y Zhifei L Yue M. Dual immune checkpoint inhibitors or combined with anti-VEGF agents in advanced, unresectable hepatocellular carcinoma. Eur J Intern Med. (2023) 111. doi: 10.1016/j.ejim.2022.12.025 36588054

[29] Riad S Daneng L Nicolas S Sairy H Wendy V Beiying D. Characterization of response to atezolizumab + bevacizumab versus sorafenib for hepatocellular carcinoma: Results from the IMbrave150 trial. Cancer Med. (2021) 10(16). doi: 10.1002/cam4.4090 PMC8366100 34189869

[30] Sorensen A Emblem K Polaskova P Jennings D Kim H Ancukiewicz M. Increased survival of glioblastoma patients who respond to antiangiogenic therapy with elevated blood perfusion. Cancer Res. (2012) 72(2):402–7. doi: 10.1158/0008-5472.CAN-11-2464 PMC3261301 22127927

[31] Yasuda S Sho M Yamato I Yoshiji H Wakatsuki K Nishiwada S. Simultaneous blockade of programmed death 1 and vascular endothelial growth factor receptor 2 (VEGFR2) induces synergistic anti-tumour effect *in vivo* . Clin Exp Immunol. (2013) 172 500–6. doi: 10.1111/cei.12069 PMC3646450 23600839

[32] Wu F Xu P Chow A Man S Krüger J Khan K. Pre- and post-operative anti-PD-L1 plus anti-angiogenic therapies in mouse breast or renal cancer models of micro- or macro-metastatic disease. Br J Cancer. (2019) 120(2):196–206. doi: 10.1038/s41416-018-0297-1 30498230 PMC6342972

[33] Joyce EO Dmitry IG Gregory DS Ekaterina K Kelly SP Sorena N. VEGF inhibits T-cell development and may contribute to tumor-induced immune suppression. Blood. (2003) 101(12). doi: 10.1182/blood-2002-07-1956 12586633

[34] Abdulaali RA Ali M Marion S Brian LE Ivo A. Potential immune-related adverse events associated with monotherapy and combination therapy of ipilimumab, nivolumab, and pembrolizumab for advanced melanoma: A systematic review and meta-analysis. Front Oncol. (2020) 10. doi: 10.3389/fonc.2020.00091 PMC7033582 32117745

[35] Johnson D Fehrenbacher L Novotny W Herbst R Nemunaitis J Jablons D. Randomized phase II trial comparing bevacizumab plus carboplatin and paclitaxel with carboplatin and paclitaxel alone in previously untreated locally advanced or metastatic non-small-cell lung cancer. J Clin Oncol. (2023) 41(13):2305–12. doi: 10.1200/JCO.22.02543 37126944

[36] Sandler A Schiller J Gray R Dimery I Brahmer J Samant M. Retrospective evaluation of the clinical and radiographic risk factors associated with severe pulmonary hemorrhage in first-line advanced, unresectable non-small-cell lung cancer treated with Carboplatin and Paclitaxel plus bevacizumab. J Clin Oncol. (2009) 27(9):1405–12. doi: 10.1200/JCO.2008.16.2412 PMC3527732 19224857

[37] Kamba T Tam B Hashizume H Haskell A Sennino B Mancuso M. VEGF-dependent plasticity of fenestrated capillaries in the normal adult microvasculature. Am J Physiol Heart Circ Physiol. (2006) 290(2):H560–76. doi: 10.1152/ajpheart.00133.2005 16172168

[38] Dardik R Livnat T Seligsohn U. Variable effects of alpha v suppression on VEGFR-2 expression in endothelial cells of different vascular beds. Thromb Haemost. (2009) 102(5):975–82. doi: 10.1160/th08-10-0687 19888537

[39] Davis E Salem J Young A Green J Ferrell P Ancell K. Hematologic complications of immune checkpoint inhibitors. Oncologist. (2019) 24(5):584–8. doi: 10.1634/theoncologist.2018-0574 PMC6516131 30819785

[40] Samantha DF Colton M Kristin E Kunal A Amar HK. Immune checkpoint inhibitor-associated thrombotic thrombocytopenic purpura in a patient with metastatic non-small-cell lung cancer. Cureus. (2021) 13(6). doi: 10.7759/cureus.16035 PMC8321598 34345535

[41] Jill ES Lindsey AF Joanne K Stacy P Daniel WO Shane EB. Antiplatelet and wound healing implications of immunotherapy and targeted cancer therapies in the perioperative period. Anesthesiology. (2023) 139(4). doi: 10.1097/ALN.0000000000004669 37698434

[42] Hsu C Rimassa L Sun H Vogel A Kaseb A. Immunotherapy in hepatocellular carcinoma: evaluation and management of adverse events associated with atezolizumab plus bevacizumab. Ther Adv Med Oncol. (2021) 13 17588359211031141. doi: 10.1177/17588359211031141 34377156 PMC8327224

[43] Gu T Jiang A Zhou C Lin A Cheng Q Liu Z. Adverse reactions associated with immune checkpoint inhibitors and bevacizumab: A pharmacovigilance analysis. Int J Cancer. (2023) 152(3):480–95. doi: 10.1002/ijc.v152.3 36274626

[44] Xia C Dong X Li H Cao M Sun D He S. Cancer statistics in China and United States, 2022: profiles, trends, and determinants. Chin Med J (Engl). (2022) 135(5):584–90. doi: 10.1097/CM9.0000000000002108 PMC8920425 35143424

[45] Cao M Li H Sun D He S Yu Y Li J. Cancer screening in China: The current status, challenges, and suggestions. Cancer Lett. (2021) 506 120–7. doi: 10.1016/j.canlet.2021.02.017 33684533

[46] Liyuan H Shenghao L Fanghang Y Hengyi W Yuxin Z Xiaoyi Z. The current status and future of targeted-immune combination for hepatocellular carcinoma. Front Immunol. (2024) 15. doi: 10.3389/fimmu.2024.1418965 PMC11330771 39161764

